# Personals

**Published:** 1890-10

**Authors:** 


### PERSONALS.

Dr. F. W. McRae and family spent the greater part of September in New York.

Dr. Virgil O. Hardon returned from the North the 1st of September.

Dr. H. V. M. Miller has returned to the city, having spent the summer at Tallulah Falls.

Dr. John C. Olmstead has been elected to the Chair of Physiology in the Southern Medical College, left vacant by the death of Dr. R. C. Word.

